# Single-virus tracking reveals variant SARS-CoV-2 spike proteins induce ACE2-independent membrane interactions

**DOI:** 10.1126/sciadv.abo3977

**Published:** 2022-12-09

**Authors:** Shaun M. Christie, Takuya Tada, Yandong Yin, Amit Bhardwaj, Nathaniel R. Landau, Eli Rothenberg

**Affiliations:** ^1^ Department of Biochemistry and Molecular Pharmacology, New York University Grossman School of Medicine, New York, NY, USA.; ^2^ Department of Microbiology, New York University Grossman School of Medicine, New York, NY, USA.

## Abstract

Severe acute respiratory syndrome coronavirus 2 (SARS-CoV-2) became a global health crisis after its emergence in 2019. Replication of the virus is initiated by binding of the viral spike (S) protein to human angiotensin-converting enzyme 2 (ACE2) on the target cell surface. Mutations acquired by SARS-CoV-2 S variants likely influence virus-target cell interaction. Here, using single-virus tracking to capture these initial steps, we observe how viruses carrying variant S interact with target cells. Specificity for ACE2 occurs for viruses with the reference sequence or D614G mutation. Analysis of the Alpha, Beta, and Delta SARS-CoV-2 variant S proteins revealed a progressive altered cell interaction with a reduced dependence on ACE2. Notably, the Delta variant S affinity was independent of ACE2. These enhanced interactions may account for the increased transmissibility of variants. Knowledge of how mutations influence cell interaction is essential for vaccine development against emerging variants of SARS-CoV-2.

## Teaser

Mutations within SARS-CoV-2 spike variants increase potential for transmissibility by altering target cell interaction.

## INTRODUCTION

Severe acute respiratory syndrome coronavirus 2 (SARS-CoV-2), the virus responsible for the current global coronavirus disease 2019 pandemic, has caused more than 529 million infections and 6.2 million deaths (as of 8 June 2022) (*1*–*3*). Coronaviridae are enveloped, single-stranded, and positive sense RNA viruses that use a spike (S) glycoprotein to interact with surface-exposed membrane proteins on target cells. S mediates cell entry by attachment to angiotensin-converting enzyme 2 (ACE2), a mammalian single-pass transmembrane receptor (*4*). SARS-CoV-2 S, Wuhan reference sequence, is 76% similar to SARS-CoV S and binds ACE2 with comparable affinity; however, novel mutations influence this interaction (*5*). Early in the pandemic, the D614G mutation was introduced, increasing transmissibility and outcompeting virus with the reference S sequence during cocirculation (*6*–*8*). D614G, which is the S2 subunit rather than the ACE2-binding interface, influences trimer conformation and infectivity (*9*). As global caseloads increased, additional S protein mutations emerged, leading to the variants of concern (Alpha, Beta, and Delta). The Alpha (B.1.1.7) and Beta (B.1.351) variants were first detected and sequenced in the United Kingdom and South Africa, respectively, emerging independently with high case numbers in mid-to-late 2020 (*10*, *11*). The Delta (B.1.617.2) variant was first identified in India and became a dominant global circulating variant (*12*). Overall, these variants display increased transmissibility attributed to various factors, including binding to ACE2 with higher affinity, evading immune recognition, and new interaction partners at the cell membrane. While infectivity assays of SARS-CoV-2 S variants report increased transmissibility, current vaccines and neutralizing antibodies are predicted to provide adequate protection from severe symptoms and hospitalization, independent of the variant (*13*–*17*). These results are important in showing how new variants influence infection in a broad sense but lack key mechanistic details of the initial virus-target cell interaction.

Single-particle tracking has been implemented for studying various viral particles, such as influenza and bacteriophage, to monitor individual virus behavior and obtain quantitative information regarding the dynamics of virus-target cell interaction in real time (*18*–*20*). Here, we used multicolor total internal reflection fluorescence (TIRF) microscopy in conjunction with single-virus tracking to determine initial interactions of pseudo-typed viral particles (PVPs) with target cells as a function of variant S mutations and ACE2. We categorized SARS-CoV-2 S interactions based on single-virus trajectories as compared to PVPs with nonspecific interaction or broad host affinity, confirming that D614G designates specificity for an ACE2-dependent target interaction. The Alpha and Beta variant S enhanced interactions with less dependence on ACE2. Superresolution microscopy (STORM) was used to map PVP colocalization with ACE2 and neuropilin-1 (Nrp1), revealing increased utilization of Nrp1 and reduced interaction with ACE2 alone by the Beta variant S. Unexpectedly, PVPs with the Delta variant S exhibit enhanced cell surface retention independent of ACE2, with individual PVPs immediately confined at the cell surface, corresponding to a high-affinity interaction. Our study provides a framework for understanding the role of SARS-CoV-2 S mutations on the initial interaction of the virus with the target cell, which is valuable in the development of novel therapeutic strategies for current and possible future outbreaks.

## RESULTS

**Fig. 1.**
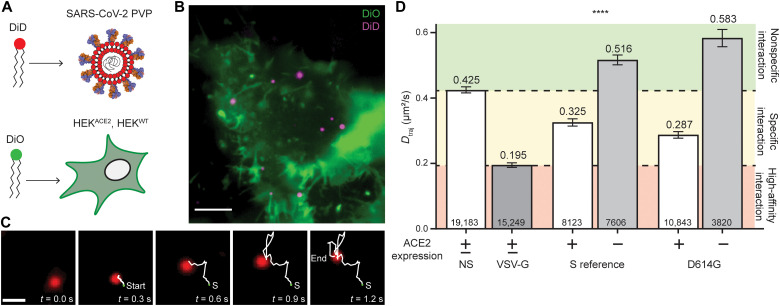
Single-virus tracking indicates that D614G enhances interaction with target cells. (**A**) PVPs are labeled with DiD and HEK cells are labeled with DiO to observe the lipid envelope/membrane. (**B**) Example TIRF image of DiO-labeled HEK cells with DiD-labeled PVPs. Scale bar, 5 μm. (**C**) Detected particles are subjected to a multiple hypothesis tracking algorithm. An example PVP is shown here, 5 of 50 frames, with detected trajectory trailing. Scale bar, 1 μm. (**D**) *D*_traj_ for particles that have a trajectory of at least eight frames. The green area represents nonspecific diffusion, the yellow area represents specific interaction diffusion (*D*_NS_ > *D*_traj_ > *D*_VSV-G_), and the red area represents high-affinity interaction. ****P < 0.0001 in all pairwise comparisons. Trajectory sample size, from at least two independent experiments, is indicated at the bottom of each bar. Error bars represent SE.

To characterize the early events of SARS-CoV-2 virus-target cell interaction, we used single-virus tracking to monitor individual PVPs and resolve their diffusion dynamics and association with the cell membrane (Fig. 1, A to C). Briefly, the envelope of SARS-CoV-2 S variant PVPs was fluorescently labeled with DiD, and particles were added to DiO-labeled wild-type human endothelial kidney (HEK^WT^) cells or those stably expressing human ACE2 (HEK^ACE2^; Fig. 1A). We used multicolor TIRF microscopy to image the movement of individual PVPs at the cell membrane (Fig. 1B). The acquired movies were subsequently analyzed via single-particle detection and tracking, followed by a trajectory quantification routine providing the specific characteristics of single-virus trajectories (Fig. 1C), including PVP diffusion (see Materials and Methods) (*21*–*23*). To determine the specific effect of SARS-CoV-2 S variants on the virus-target cell interaction, we established a normalized scale based on the diffusion characteristics of two types of PVPs that model distinct modes of interaction: nonspecific interactions toward cells using PVPs lacking viral S proteins (NS) and broad cell interactions using PVPs with the vesicular stomatitis virus glycoprotein (VSV-G), which recognizes low-density lipoprotein receptor (*13*, *24*). The measured diffusion coefficients of both NS and VSV-G PVPs were independent of ACE2 expression (fig. S1).

### D614G increases affinity for host cells dependent on ACE2

SARS-CoV-2 S with the D614G mutation, first described by Korber *et al.* (*6*), became the dominant global variant between March and May 2020. Initial evidence suggested that the mutation allowed for higher infectivity and transmissibility, as well as outcompeting the S reference sequence during cocirculation (*7*, *8*). To determine the influence of D614G on the interaction with target cells, we collected single-virus trajectories on HEK^ACE2^ and HEK^WT^ cells for PVPs with the reference sequence or D614G S proteins. Average trajectory diffusion coefficient, *D*_traj_ (Fig. 1D), was quantified from the mean squared displacement (MSD) of all trajectories with durations longer than eight frames (>240 ms), thereby avoiding transiently interacting particles (see Materials and Methods) (*21*–*23*). The diffusion values obtained for NS and VSV-G controls (0.425 ± 0.009 and 0.195 ± 0.007 μm^2^/s, respectively) provide a calibration scale for categorizing the magnitude of *D*_traj_ into three ranks of membrane interaction: (i) diffusion under nonspecific interaction (*D*_traj_ > *D*_NS_), (ii) diffusion under specific protein-protein interaction (*D*_NS_ > *D*_traj_ > *D*_VSV-G_), and (iii) diffusion under specific, high-affinity interaction (*D*_VSV-G_ > *D*_traj_). PVPs with SARS-CoV-2 reference sequence S display characteristic *D*_traj_ corresponding to a specific membrane protein interaction with HEK^ACE2^ cells. Introduction of D614G in the S protein resulted in reduced *D*_traj_ corresponding to an increase in its interaction with HEK^ACE2^ cells. Moreover, we found that the magnitude of diffusion is strictly dependent on ACE2, as analysis of trajectories for both PVPs on HEK^WT^ cells resulted in an increase in *D*_traj_ to the range corresponding to nonspecific interactions. Combined, these results demonstrate that the D614G mutation allows for increased interaction with HEK^ACE2^ cells and that the interaction is highly dependent on ACE2.

### Alpha and Beta variant S further enhances target cell interaction with reduced ACE2 dependence

**Fig. 2.**
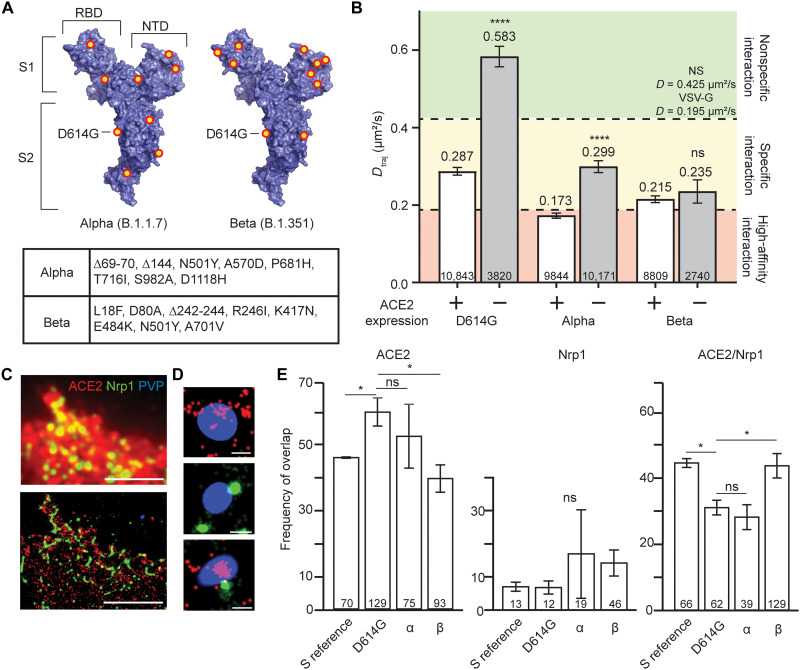
Globally circulating variants interact with host cells in an ACE2-independent manner. (**A**) Mutations and deletions (red/yellow dots) for the Alpha (left) and Beta (right) variants of SARS-CoV-2 S monomer [Protein Data Bank (PDB) 6VXX] (*52*). Inset table lists the mutations for each variant. (**B**) *D*_traj_ for trajectories greater than eight frames. Colored areas represent the same control ranges from Fig. 1D. ****P < 0.0001. Trajectory sample size, from at least two independent experiments, is indicated at the bottom of each bar. (**C**) Representative epifluorescence image (top) and corresponding reconstructed STORM image (bottom) of HEK^ACE2^ cells immunostained for ACE2 (red) and Nrp1 (green) incubated with DiO-labeled PVPs (blue). Scale bars, 5 μm. (**D**) Representative magnified STORM/TIRF images of single PVPs colocalized with ACE2 (top), Nrp1 (middle), or ACE2/Nrp1 (bottom). Scale bars, 200 nm. Average full width at half maximum of PVPs is 275 nm (fig. S3A). Additional representative magnified particles can be found in fig. S3B. (**E**) PVPs categorized into populations according to (D) and calculated as percent of the total observed particles. *P < 0.05. PVP sample size, from at least two independent experiments, is indicated at the bottom of each bar. Particles were extracted from the following number of individual cells; S reference, 23; D614G, 21; α, 20; β, 31. Error bars represent SE. ns, not significant.

With the continued global spread of SARS-CoV-2, additional variants with residue changes in the S1 and S2 S subunits emerged independent of one another but include the D614G mutation (*10*, *11*). Alterations to the receptor binding domain (RBD) likely influence ACE2 affinity, while those in the N-terminal domain (NTD) play a role in antibody evasion. The Alpha variant (B.1.1.7), first reported in the United Kingdom in December 2020 (*10*), contains seven point mutations (including D614G) and two deletions as compared to the reference sequence S (Fig. 2A, left). The Beta variant (B.1.351), first reported in South Africa in December 2020 (*11*), contains eight point mutations (including D614G) and one deletion as compared to the reference sequence S (Fig. 2A, right). To determine how these mutations affect target cell interaction, we quantified the *D*_traj_ of PVPs for each variant on HEK^WT^ and HEK^ACE2^ cells (Fig. 2B). For HEK^ACE2^ cells, *D*_traj_ of both Alpha and Beta PVPs is greatly decreased from that of D614G, with the Alpha variant S *D*_traj_ falling within the range corresponding to high-affinity interactions. Notably, when incubated with HEK^WT^ cells, *D*_traj_ does not increase to the range for nonspecific interaction for either Alpha or Beta as observed for D614G. While Alpha variant S does have an increased *D*_traj_, this does not significantly differ from D614G on HEK^ACE2^ cells, indicating high affinity for ACE2 but an independence from ACE2 interaction as the cause for slow dynamics on HEK^WT^ cells. *D*_traj_ for the Beta variant S is not significantly changed under HEK^WT^ cell conditions, suggesting that the strong virus-target cell interaction is independent of ACE2.

While ACE2 is the primary receptor for SARS-CoV-2, alternative entry cofactors have been identified, as was the case for SARS-CoV and Middle East respiratory syndrome coronavirus (*25*). Nrp1, which is related to complex signaling with vascular endothelial growth factor receptor and plexins in many cell types, has been implicated as a co-receptor for SARS-CoV-2 S (*26*–*30*). To evaluate the role of Nrp1 in target cell interactions, we used STORM, which provides enhanced detection sensitivity and localization precision of several nanometers for quantifying the colocalization of individual PVPs with ACE2 and Nrp1 in cells (*31*–*33*). STORM imaging shows the detailed features of localized ACE2 or Nrp1 that are otherwise masked in diffraction-limited epifluorescence (see Fig. 2C and Materials and Methods). We imaged HEK^ACE2^ cells that were fixed and immunofluorescently labeled for ACE2 and Nrp1 after 1 hour of incubation with PVPs of each S variant. To determine whether the variant S affects PVP colocalization, we categorized the overlayed PVPs into populations that colocalized with ACE2 alone, with Nrp1 alone, or with both receptors (Fig. 2D). SARS-CoV-2 S reference sequence PVPs had no significant interaction with Nrp1 alone but had equal amounts of PVPs interacting with ACE2 alone or with both ACE2 and Nrp1 (Fig. 2E). D614G mutation’s enhanced ACE2 affinity is noted by reduced interaction with both receptors and increased colocalization with ACE2 alone. A shift toward ACE2 independence is demonstrated by the Alpha variant S variability in colocalization with each receptor alone, while the Beta variant S showed a consistent decrease in colocalization with ACE2 alone and increases in populations localized with Nrp1 alone and both receptors. These colocalization trends are consistent across time points (fig. S2). Combined results from *D*_traj_ analysis and STORM imaging demonstrated that both the Alpha and Beta variants shift toward an ACE2-independent interaction with the target cell and, in the case of Beta, use Nrp1 for the interaction.

### The Delta variant exhibits ACE2-independent host interactions with high affinity and membrane confinement

**Fig. 3.**
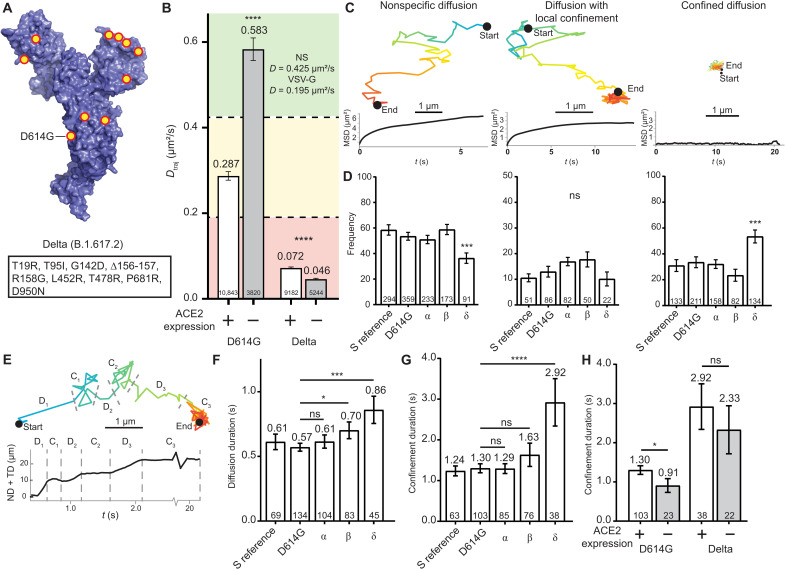
The Delta variant produces a strong ACE2-independent interaction and exhibits reduced motion at the cell surface. (**A**) Mutations and deletions (red/yellow dots) for the Delta variant of the SARS-CoV-2 monomer (PDB 6VXX) (*52*). Inset table lists the mutations. (**B**) *D*_traj_ for trajectories greater than eight frames. Colored areas represent the same control ranges from Fig. 1D. ****P < 0.0001. Trajectory sample size, from at least two independent experiments, is indicated at the bottom of each bar. (**C**) Representative trajectories and MSD curves for *D*_N_ (left), *D*_LC_ (middle), and *D*_C_ (right). (**D**) Percentage of trajectory population in each classification. ***P < 0.001. (**E**) Top: Representative *D*_LC_ trajectory with dotted lines indicating transition points between states. Bottom: Representative sum of the net and total displacement, with a rolling average of five frames. (**F**) Average diffusion durations for each variant PVP. *P < 0.05 and ***P < 0.001. (**G**) Average confinement durations for each variant PVP. ****P < 0.0001. (**H**) Comparison of confinement durations of D614G and Delta variant S for HEK^ACE2^ and HEK^WT^ cells. *P < 0.05 Trajectory sample size, from at least two independent experiments, is indicated at the bottom of each bar. Error bars represent SE.

As of mid-2021, the Delta variant, first identified in India, became the most prevalent variant, displaying 50% higher transmissibility and outcompeting the Alpha variant (*12*). The variant contains eight point mutations (plus D614G) and one deletion (Fig. 3A). To evaluate how these mutations affect interaction with target cells, we measured the dynamics of PVPs for the variant S on HEK^WT^ and HEK^ACE2^ cells and quantified *D*_traj_ (Fig. 3B). The Delta variant S exhibits a decrease in *D*_traj_ as compared to D614G, resulting in diffusion behavior that is well within the range corresponding to high-affinity interaction. Unexpectedly, the *D*_traj_ for Delta variant PVPs measured with HEK^WT^ cells remained in the range for high-affinity interaction, displaying a further reduction as compared to *D*_traj_ on HEK^ACE2^ cells. This demonstrates that a high affinity for ACE2 is not required for the strong interaction of the Delta variant S with target cells. To examine whether the interaction of the Delta S arises from increased utilization of Nrp1, we analyzed the colocalization of PVPs via STORM imaging, which showed a colocalization frequency similar to D614G. Combined, these results indicate that the high affinity of the Delta variant S is due to interaction with factors in the membrane other than ACE2 and Nrp1 (fig. S4).

To gain further mechanistic insight into the interaction leading to the substantial decrease in *D*_traj_ of the Delta variant, we conducted further characterization and classification of single-virus trajectories, examining trajectories with >40 frames (>1.2 s) after an initial membrane landing event. Individual PVPs displayed several characteristic diffusion behaviors, shown in the representative trajectories and corresponding MSD curves in Fig. 3C, which we attributed to diffusion from three different classes of membrane interaction—two-dimensional (2D) diffusion under nonspecific interactions (*D*_N_ > 0.1 μm^2^/s), confined diffusion under localized interaction (0.1 > *D*_LC_ > 0.01 μm^2^/s), and locally bound particles with limited diffusion (*D*_C_ < 0.01 μm^2^/s)—and quantified the frequency of each trajectory classification for all PVPs (Fig. 3D). The percentage of PVPs with *D*_N_ and *D*_C_ are relatively consistent for PVPs with SARS-CoV-2 S reference sequence, D614G, and Alpha and Beta variants. Slight differences occur in the rate of *D*_LC_ where each variant S displays increased probability of this intermediate process. The Delta variant S, however, shows a reduction in *D*_N_ along with an increase in *D*_C_ (53% of particles), in agreement with the reduction of *D*_traj_. An endpoint infectivity assay for each PVP variant demonstrates increasing infection of HEK^WT^ cells but with a 100-fold increase in HEK^ACE2^ cells (fig. S5). These results indicate high-affinity, ACE2-independent interaction and capture of the PVPs, which will inevitably lead to ACE2-dependent infection at a higher rate than those viruses that may dissociate during *D*_N_ and *D*_LC_ processes (*34*).

Although *D*_LC_ events were a rare event for PVPs, we wanted to characterize whether the Delta variant S caused fundamental differences in this intermediate step of virus-target cell interaction. The sum of the net and total displacements was used to determine switches between diffusing and confined portions of the PVP trajectory (Fig. 3E). When diffusing, the sum will increase over time and plateau during regions of confinement, and we quantified the duration of each trajectory segment. *D*_N_ durations are relatively unchanged between variants with increases occurring for Beta and Delta variants (Fig. 3F). This can indicate that weak-affinity interactions keep PVPs near the cell surface rather than diffusing away. Meanwhile, *D*_C_ durations of trajectories are unaltered, except in the case of the Delta variant S, which are increased (Fig. 3G). This demonstrates that when the Delta variant PVPs are not immediately confined, there is an increased chance of establishing high-affinity interactions. These interactions are likely ACE2 independent, as the *D*_C_ durations of Delta S PVPs are not significantly decreased on HEK^WT^ cells (Fig. 3H). Each result for the Delta variant adds to the evidence that selective pressure provides S with a path toward ACE2-independent and high-affinity interactions.

## DISCUSSION

The continued global spread of SARS-CoV-2 provides a setting for the emergence of variants with novel mutations in S (*35*). Selective pressure has resulted in viral S with higher affinity for target cell interaction and with mutations that allow for escape from immune recognition (*5*, *10*–*16*). Understanding how mutations in S can influence initial events in virus-target cell interaction will provide insight on transmission and pathogenesis, which allows for the development of improved therapeutics and vaccines in current and future virus outbreaks. The use of single-virus tracking allowed us to determine changing dynamics as the virus mutates and understand how the interaction with ACE2 is altered. The D614G mutation has an increased ACE2 affinity, as demonstrated by reduced *D*_traj_, and allows for ACE2-independent target cell binding, as PVPs incubated on HEK^WT^ cells have *D*_traj_ > *D*_NS_ (Fig. 1D). This conclusion is in agreement with structural and functional studies of the effect of D614G on increased transmissibility, although the mutation is not in the RBD motif (*6*–*9*). Endpoint infectivity assays also show that infection is increased in an ACE2-dependent manner (*9*, *13*). Structural analysis by cryo–electron microscopy suggests that the mutation changes the open-closed conformation of the RBD, preferentially displaying the RBD with the ACE2-binding motif exposed, enhancing virus-target cell interaction (*9*).

As the number of mutations in S is increased, ACE2 dependence is reduced; here, the Alpha and Beta variant S allows for increased interaction with HEK^WT^ cells. Mutations in the RBD and NTD could allow for ACE2 independence by enhancing an interaction with a secondary receptor, such as Nrp1, which contains an interaction site for the S protein. This interaction was demonstrated by STORM imaging at various time points following virus introduction where interaction with Nrp1 is enhanced but reduced with ACE2 alone (Fig. 2E and fig. S2). Whether this is caused by mutations in the RBD or at other sites that change conformation, such as the effect seen with D614G, is unknown. The cytoplasmic domain of ACE2 is not required for virus entry, adding to evidence that co-receptors may be necessary for intracellular signaling (*36*).

**Fig. 4.**
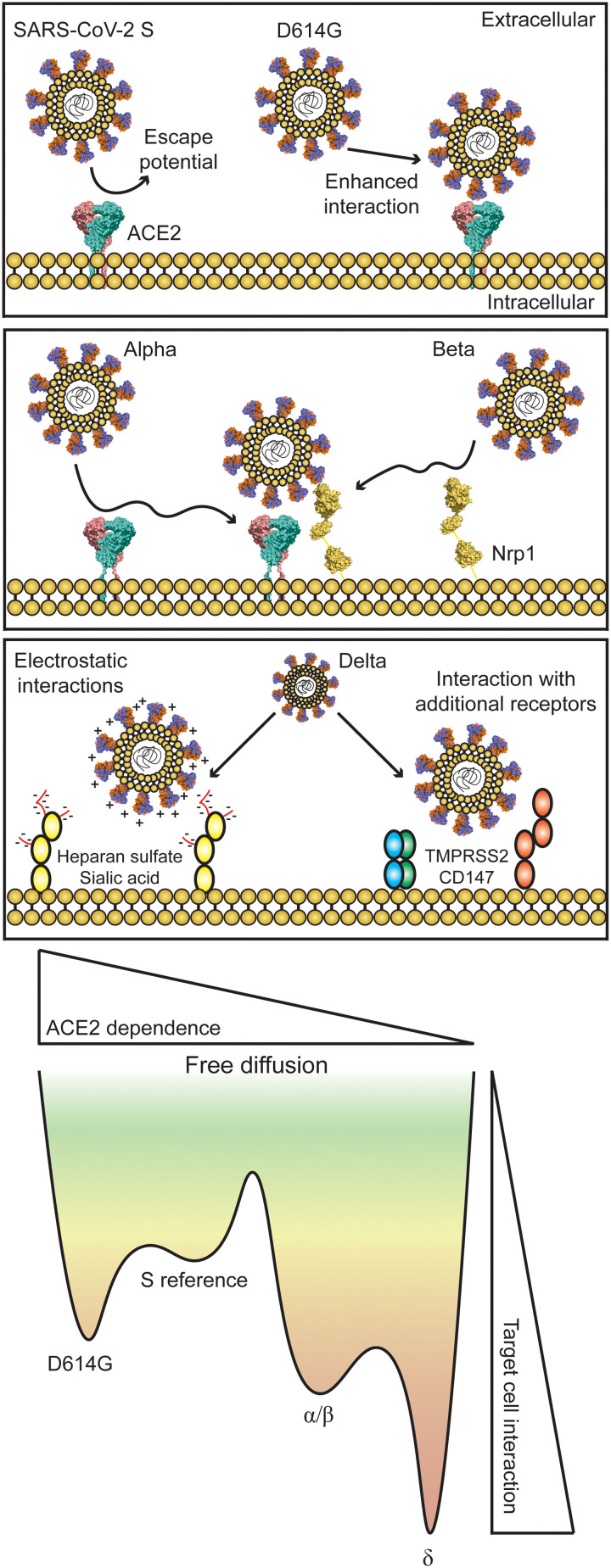
Theoretical model of virus-target cell interaction influenced by variant emergence. SARS-CoV-2 S displays affinity for the ACE2 receptor [PDB 6M18 (*53*)] but has the probability of escape during initial virus-target cell interactions. D614G displays increased interaction and decreased escape potential dependent on ACE2. Alpha and Beta also have increased interaction but exhibit reduced ACE2 dependence. Nrp1 [PDB 4GZ9 (*54*) and 5L73 (*55*)] consistently colocalizes with ACE2 and Beta variant PVPs and could be involved with initial interaction. The Delta variant displays characteristics of ACE2 independence with a high probability of confinement at the cell surface.

Last, the emergence of the Delta variant created another wave of infections with higher transmissibility (*12*, *15*). A shift toward ACE2 independence is observed by reduced *D*_traj_ in HEK^ACE2^ and HEK^WT^ cells, and measured trajectories demonstrate the increased likelihood of confining PVPs at the cell surface even in the absence of ACE2 (Fig. 3). While the influence of Nrp1 is less clear for this variant, additional research has shown SARS-CoV-2 S affinity toward several potential receptors, such as furin-like proteases necessary for viral cleavage/fusion, which could cause the change in dynamics related to the Delta variant as they are expressed in this model cell line (*5*, *37*–*41*). A large number of studies have used lentiviral PVPs under the assumption that they act in a consistent manner with authentic virus particles, but we cannot rule out that small amounts of cell surface protein may also be incorporated into the PVPs, influencing virion attachment (*13*). Changes in S glycosylation may also influence the interaction with cellular glycocalyx. Heparan sulfates and sialic acids play a role in SARS-CoV-2 and other virus attachment, with many potential binding sites across S (*42*, *43*). Multiple arginine point mutations in the Delta variant S could allow for increased electrostatic interactions of S with these moieties at the cell surface (*44*). This was observed for SARS-CoV and SARS-CoV-2 where threonine to lysine and glutamine to asparagine enhance the electropositive surface of S (*42*). A theoretical model of these observations is presented in Fig. 4.

As novel SARS-CoV-2 variants with mutations in S continue to arise, there is the possibility that a more aggressive variant will emerge with increased pathogenicity and evasion of adaptive immune response. A better understanding of how S mutations influence virus-target cell interaction will provide insight for the development of novel therapeutics and vaccines against current and future viral outbreaks.

## MATERIALS AND METHODS

### Production of SARS-CoV-2 S mutations and variants

Codon-optimized SARS-CoV-2 S gene (Wuhan-Hu-1/2019) was chemically synthesized by Thermo Fisher Scientific and cloned into pcDNA6 (Invitrogen). To generate pcCOV2-Δ19S, S gene was amplified with a forward and reverse primer that deleted the 19 C-terminal amino acids. The amplicon was cloned into the Kpn I and Xho I sites of pcDNA6. Point mutations in variant S plasmids (D614G, Alpha, Beta, and Delta) were introduced by overlap extension polymerase chain reaction (PCR). All sequences were confirmed by DNA nucleotide sequence analysis. Lentiviral packaging plasmids pMDL and pRSV.Rev were previously described (*14*).

### PVP production

Rather than using clinical SARS-CoV-2 samples for these studies, pseudo-typed lentiviral particles harboring S and its variants were produced under Biosafety Level 2 (BSL-2) conditions. SARS-CoV-2 S or VSV-G pseudo-typed lentiviral stocks were produced by cotransfecting HEK^WT^ cells (accession number CRL-1573) with pMDL, pLenti.GFP-NLuc, pcCoV2.S-Δ19 (or variants thereof), and pRSV.Rev by calcium phosphate coprecipitation (*13*). Two days after transfection, virus-containing supernatant was harvested and concentrated by ultracentrifugation on a 20% sucrose cushion at 30,000 rpm for 90 min. The virus pellet was resuspended in Dulbecco’s modified Eagle’s medium (DMEM)/10% fetal bovine serum and frozen in aliquots at −80°C. Virus titers were measured by green fluorescent protein (GFP)–positive cell populations on HEK^ACE2^ cells by flow cytometry. Alternatively, virus titers were measured by reverse transcriptase activity using a real-time PCR assay (*45*). As noted in prior publications, which use these PVPs, S protein incorporation into PVPs is consistent across variant S proteins (*14*, *46*).

### Cell culture

The HEK cell line was adopted in our study since it is a well-established model system that is broadly used for SARS-CoV-2 infectivity assays, but to further verify our results, we also monitored control viral trajectories in BEAS-2B (accession number CVCL-0168), a nontumorigenic bronchial epithelial line, where trends are consistent for both cell lines (fig. S6). BEAS-2B cells were cultured in Bronchial Epithelial Basal Media (BEBM) and Bronchial Epithelial Growth Media (BEGM) BulletKit media (Lonza, CC-3170) supplemented with 1% penicillin-streptomycin. Culture surfaces for this cell line were coated with a solution of fibronectin (0.01 mg/ml), bovine collagen type I (0.03 mg/ml), and bovine serum albumin (BSA; 0.01 mg/ml) in complete media overnight at 37°C before plating cells. HEK^WT^ cells were made to stably express human ACE2 by transfection of cells with pLenti.ACE2-HA using Lipofectamine 2000 (Invitrogen). Two days after transfection, the cells were cloned at limiting dilution in puromycin (1 μg/ml). ACE2-expressing single-cell clones were analyzed by flow cytometry, and a single clone was chosen. HEK^WT^ and HEK^ACE2^ cells were then cultured using standard procedures. Culture medium was consisted of DMEM supplemented with 10% fetal bovine serum and 1% penicillin-streptomycin. For HEK^ACE2^ cells, puromycin was added at a concentration of 1 μg/ml to ensure continued selection. Cells were passaged at 70 to 90% confluency by trypsinization to either 35-mm glass-bottom dishes (Mattek Corporation) for live-cell experiments or 22-mm glass coverslips (Thermo Fisher Scientific, 12-548-B) for STORM experiments.

### Live-cell imaging

Vybrant lipid interchelating dyes DiO and DiD (Invitrogen, V22886 and V22887) were used to uniformly label the cell membrane and viral envelope. HEK^WT^ or HEK^ACE2^ cells were labeled with 2 nmol of DiO in 2 ml of Fluorobrite DMEM (Thermo Fisher Scientific, A1896701) at 37°C for 10 min. Excess dye was removed by two washes with fresh imaging media at 37°C for 10 min each. Cells were used immediately after the labeling procedure. PVPs were labeled before each experiment with 2 nmol of DiD in 50 μl of virus stock at room temperature (RT) for 1 hour. Excess dye was removed by Zeba 7K desalting column (Thermo Fisher Scientific, 89883). DiD will self-quench at high concentrations, and 2 nmol/50 μl was chosen as higher concentrations reduced the number of observable particles. Particle concentration was determined by addition to a glass coverslip, imaging of 25 random areas, particle analysis in ImageJ, and input to Eq. 1 (*47*)

$$[\mathrm{Particles}]=\text{Average spots}\;*\;\;\frac{\text{Coverslip SA}}{\text{Image SA}}\;*\;\mathrm{Dilution}\;*\;\frac{1}{V}$$ (1)

PVPs were added at a concentration of 10^7^ particles/ml to labeled cells on the microscope stage. Using this quantification method with nontransfected HEK^WT^ cell media, less than 10% of labeled particles do not contain viral-loaded proteins and would be averaged out across conditions. Previous work with these PVPs establishes the high abundance of viral S proteins and viral capsid (by p-24) in each variant sample (*13*, *14*, *46*). Particles produced without viral S proteins (NS) and those with VSV-G (broad low-density lipoprotein recognition) were used to calibrate a scale of diffusion for noninteracting and high-affinity interaction, respectively (Fig. 1D). Samples were imaged on a custom-built inverted optical imaging platform based on a Leica DMI 300 inverted microscope with two laser lines, 473 nm (Laserglow Technologies, R471003FX) and 639 nm (Ultralaser, MRL-FN-639-800). Lasers were combined using dichroic mirrors and focused onto the back aperture of an oil immersion objective [Olympus, UApo N, 150×, numerical aperture (NA) = 1.45, TIRF] via multiband dichroic mirror (Semrock, FF408/504/581/667/762-Di01). To observe fluorescence at the cell membrane, samples were imaged using TIR where fluorophores are excited by incident light at the critical angle of the cell-glass interface to observe the area near the cell membrane (Fig. 1B). Observed particles diffuse between the basal cell surface and the glass coverslip, but prior studies have indicated that there are nonsignificant differences in diffusion at the apical and basal surfaces (*48*). Fluorescence emission collected back through the objective was sent to a scientific Complementary Metal-Oxide-Semicoductor (sCMOS) camera (Photometrics, Prime 95B) via a two-way emission splitter (Cairn Research, Optosplit II) using a filter set for DiO (Semrock, FF01-510/42) and DiD (Semrock, FF01-676/37) spectrally separated by a dichroic mirror (Semrock, FF552-Di01). Acquisitions consist of 1000 frames and 30 ms of duration between frames, with both channels captured simultaneously. Acquisitions were carried out for up to 30 min after PVPs began to diffuse near the cell surface. Images were then mapped to correct for chromatic aberrations using a polynomial morph-type mapping algorithm via a custom MATLAB script. Before each experiment, a calibration map was generated by imaging fluorescent beads (Tetraspeck Microspheres, 0.1 μm, Invitrogen) in each channel. A second polynomial function was optimized to fit localizations in the DiO channel to their location in the DiD channel. This function is used to map the molecule localizations of each experimental sample.

### Spot detection and spot tracking via Icy open source software

Acquired and mapped images were loaded into Icy open source software for detection and tracking of individual particles (*21*). The Spot Detector plugin was used to isolate particles in each frame running in UDWTWavelet detection mode for bright spots over dark background using scale 3 and 75% sensitivity (*23*). Tracking parameters were estimated for one image sequence in the middle of the total set of acquisitions and used for all subsequent analysis of the same PVP. The Spot Tracking plugin was used to obtain trajectory coordinates for each image sequence using multiple hypothesis tracking method (*22*). Motion Profiler processor with real units was used to determine trajectory durations and displacements. Export tracks to XLS processor was used to obtain trajectory coordinates for further processing and analysis. Using MATLAB software, diffusion coefficients were obtained by MSD analysis for trajectories longer than eight frames to have enough points for efficient calculation of diffusion and to focus on specifically interacting particles rather than those with short trajectories that quickly make contact in the illuminated area before immediately diffusing away. The number of trajectories collected per cell was relatively consistent overall, indicating that initial diffusion near the cell membrane is not dependent on viral glycoproteins, but as described in the Results and Discussion, particle dynamics are highly influenced by SARS-CoV-2 S mutation (fig. S7).

### Individual trajectory analysis

A Python graphical user interface (GUI) was created to filter and sort individual trajectories using imported trajectory coordinates. Trajectories with >40 frames (>1.2 s) were used for classification to obtain trajectories long enough to distinguish free and depressed diffusion. This is approximately double the average trajectory duration and only includes those detected after the first frame of the acquisition. This ensures that overall classification populations are not biased toward particles already present, whether immobile on the cell surface or internalized, and that trajectories are long enough to distinguish changes in local diffusion for subtrajectory analysis. Classification was based on overall trajectory quality, MSD, and diffusion. Particles were considered confined if the majority of trajectory was within ~180 nm^2^ (3 × 3 pixel). Subtrajectory analysis was carried out by observation of the change in sum of the net and total displacement to determine switch points between free and confined diffusion during trajectory.

### Stochastic optical reconstruction microscopy

HEK^ACE2^ cells were split to glass coverslips approximately 18 to 24 hours before incubation with PVPs. Here, particles are labeled in the same manner as above but with DiO rather than DiD. Coverslips with adhered cells were incubated with labeled PVPs for 15, 30, and 60 min at RT. Immediately following incubation, cells were washed with 1× phosphate-buffered saline (PBS) and fixed using a solution of 4% paraformaldehyde and 0.01% glutaraldehyde for 10 min at RT, followed by neutralization with 0.1% (w/v) NaBH_4_ in PBS for 10 min at RT. Cells were blocked overnight at 4°C using a buffer containing 2% glycine, 2% BSA, 0.2% gelatin, and 50 mM NH_4_Cl in PBS. On the day of imaging, cells were immunostained with primary antibodies for ACE2 (ab108252) and Nrp1 (DDX0440P) for 1 hour at RT, followed by secondary immunostaining with antibodies conjugated to AF647 (srbAF647-1) and AF568 (A11031), respectively, for 30 min at RT. Nrp1 is endogenously expressed by HEK cells at low but detectable levels (*49*).

Coverslips with labeled cells were mounted on glass slides with predrilled holes for imaging buffer exchange. Immediately before imaging, PBS solution is exchanged for imaging buffer containing glucose oxidase (1 mg/ml; Sigma-Aldrich, G2133), catalase (0.02 mg/ml; Sigma-Aldrich, C3155), 10% glucose (Sigma-Aldrich, G8270), and 100 mM mercaptoethylamine (Thermo Fisher Scientific, 20408) to promote triplet state blinking of fluorophores. Samples are imaged on a custom-built optical imaging platform based on a Leica DMI 300 inverted microscope with three laser lines: 488 nm (Coherent, Sapphire 488 LPX), 561 nm (Coherent, Sapphire 561 LPX), and 639 nm (Ultralaser, MRL-FN-639-1.2) (*32*). Lasers were combined using dichroic mirrors and focused onto the back aperture of an oil immersion objective (Olympus, UApo N, 100×, NA = 1.49, TIRF) via a multiband dichroic mirror (Semrock, 408/504/581/667/762-Di01). To observe fluorescence at the cell membrane, samples were excited using TIR. Fluorescence emission collected back through the objective was directed to an sCMOS camera (Photometrics, Prime 95B). Acquisitions consist of 2000 frames and 0 ms of duration between frames, with each channel captured sequentially using band-pass filters for DiO (Semrock, FF01-531/40), AF568 (Semrock, FF01-607/36), and AF647 (Semrock, FF01-676/37). A 405-nm laser line (CNI Laser, MDL-III-405-500) was used to enhance the recovery of dark-state AF647 fluorophores during acquisition. Images were acquired using Micro-Manager (v1.4) software.

In a similar manner to above, images were mapped to correct for chromatic aberrations using a polynomial morph-type mapping algorithm via a custom MATLAB script. Before each experiment, a calibration map was generated by imaging fluorescent beads in each channel. A second polynomial function was optimized to fit localizations in DiO and AF568 channels to their location in the AF647 channel (*50*). This function is used to map the molecule localizations of each experimental sample. Each frame of a raw image stack was box filtered with a box size of four times the full width at half maximum of a 2D Gaussian point spread function (PSF). Regions (7 × 7 pixel) around local maxima from all frames were submitted for 2D Gaussian multi-PSF fitting, performed by graphics processing unit (GPU), using maximum likelihood estimation algorithm. The fitting accuracy was estimated by Cramer-Rao lower bound (CRLB). Localizations that appear in consecutive frames within 2.5× the localization precision were considered as one blinking event and averaged into one localization weighted by the inverse of its own CRLB (*33*, *51*). For display purposes, the representative images were generated by rendering the raw coordinates into a 10-nm pixel canvas and convolved with a 2D Gaussian (σ = 10 nm) kernel. Each set of PVPs produced roughly the same amount of particle observations per cell (fig. S8). Because of the nonblinking nature of DiO-PVPs, the TIRF/highly inclined and laminated optical sheet microscopy image is scaled and overlaid with the reconstructed receptor image. PVPs within the cell body are isolated with a 100 × 100–pixel region of interest (ROI) to determine colocalization with either labeled receptor. The 100 × 100–pixel size ROI was chosen as approximately 4× the diameter of a diffraction-limited PVP and 10× the assumed diameter of the physical PVP (60 to 140 nm). To validate that the overlap of PVPs and receptors was not due to variations in receptor density from cell to cell or ROI to ROI, receptor density by autocorrelation and colocalization by cross-correlation was performed for a subset of reconstructed images. Figure S9A reports no significant cell-to-cell variability of receptor density, while fig. S9B indicates that randomization of one set of coordinates reduces the ACE2-Nrp1 colocalization magnitude (*33*).

## Notes

View/request a protocol for this paper from *Bio-protocol*.
