# Supplementary material for: Single-virus tracking reveals variant SARS-CoV-2 spike proteins induce ACE2-independent membrane interactions
Source: Sci Adv. 2022 Dec 9;8(49):eabo3977. doi: 10.1126/sciadv.abo3977 (PMC9733935; doi:10.1126/sciadv.abo3977)
Supplement: Supplementary file 1 — Figs. S1 to S9 [file sciadv.abo3977_sm.pdf]

Science Advances
AAAS

# Supplementary Materials for

## Single-virus tracking reveals variant SARS-CoV-2 spike proteins induce ACE2-independent membrane interactions

Shaun M. Christie *et al.*

Corresponding author: Eli Rothenberg, eli.rothenberg@nyulangone.org; Nathaniel R. Landua, nathaniel.landau@med.nyu.edu

*Sci. Adv.* **8**, eabo3977 (2022)
DOI: 10.1126/sciadv.abo3977

**This PDF file includes:**

Figs. S1 to S9

**Supplementary Figures:**

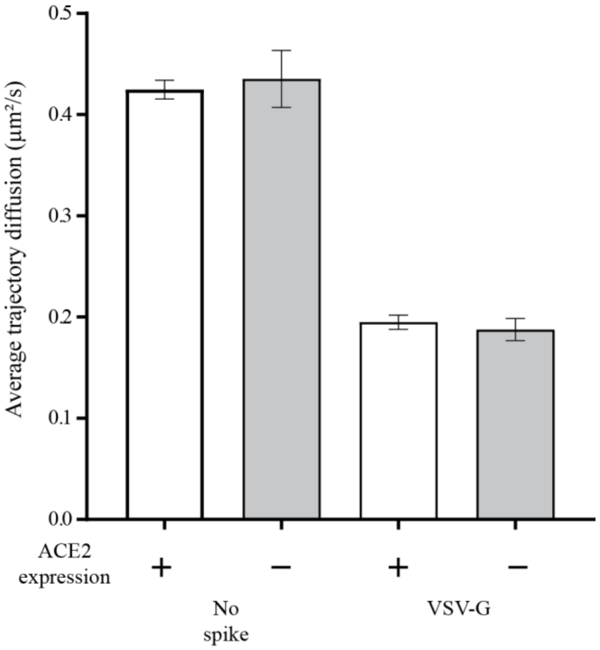


**Supplementary Figure 1:** $D_{traj}$ for NS and VSV-G PVP controls is independent of ACE2 expression

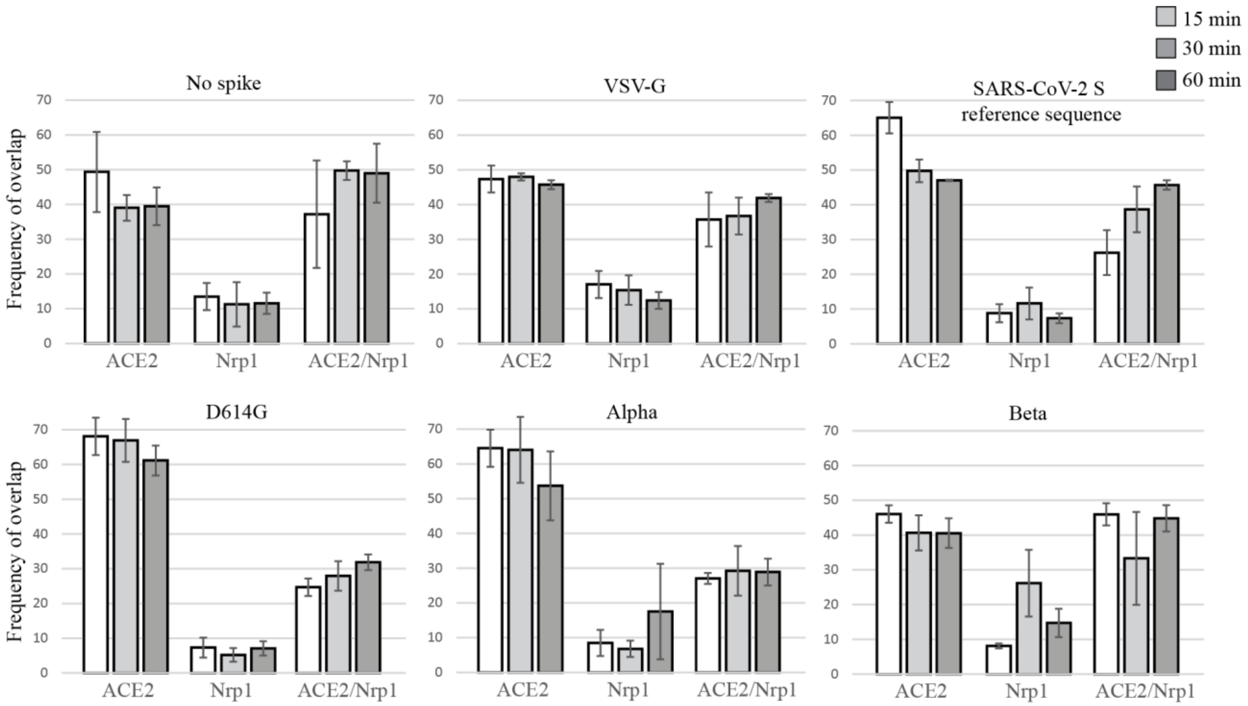

**Supplementary Figure 2:** PVP were categorized into colocalization populations based on Fig. 2D for each time point. Trends are relatively consistent for each species and time point.

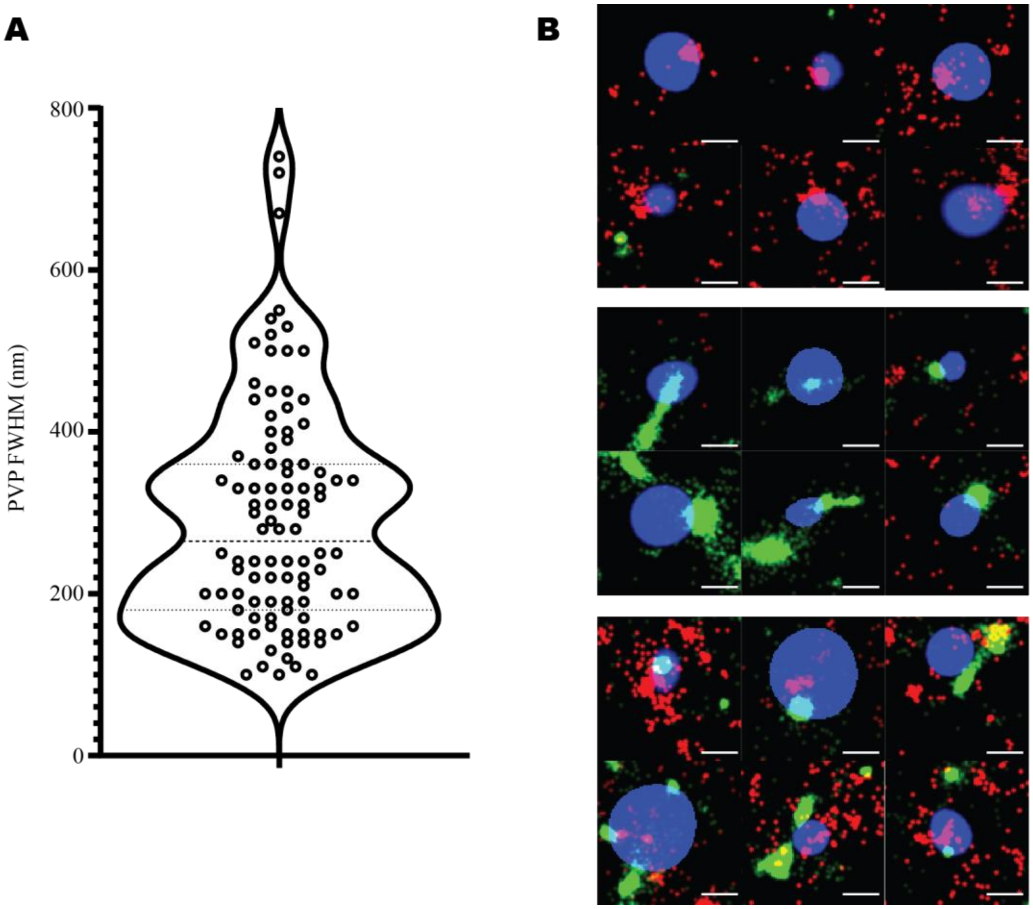


**Supplementary Figure 3:** (**A**) PVP FWHM size distribution from STORM/TIRF images. 100 random particles from different variants display an average particle size of approximately 275 nm. Distribution is not significantly different between variants. (**B**) Representative magnified STORM/TIRF images of single PVPs colocalized with ACE2 (*top*), Nrp1 (*middle*), or ACE2/Nrp1 (*bottom*). Scale bar represents 200 nm.

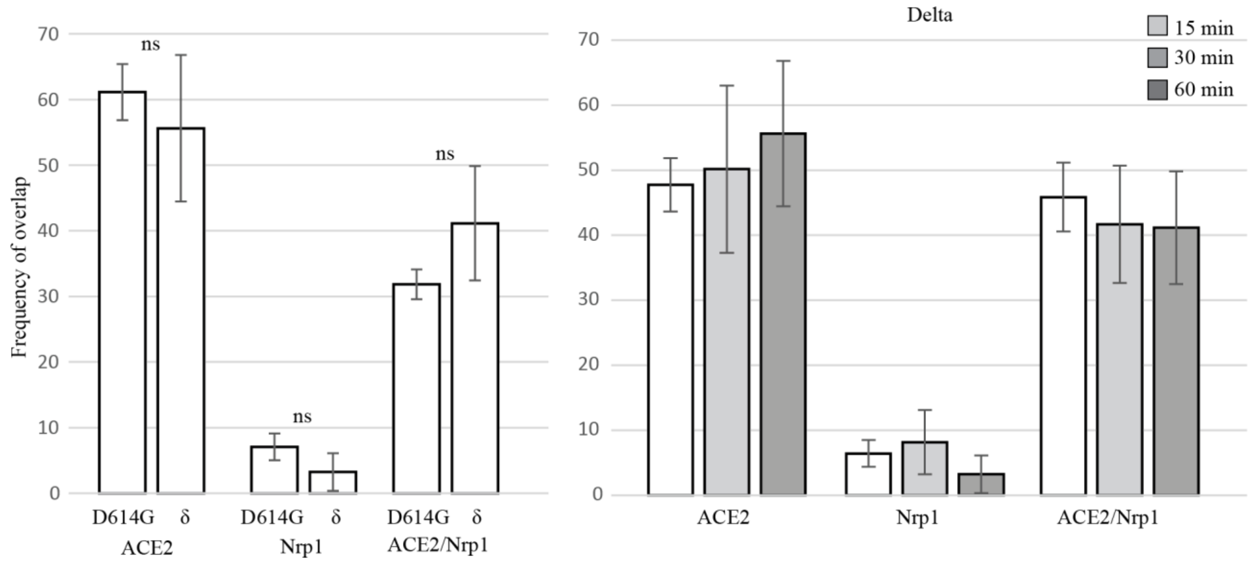


**Supplementary Figure 4:** PVPs were categorized into colocalization populations according to Fig. 2D and calculated as a percent of total observed particles. *Left* Comparison of D614G and Delta variant S STORM imaging. *Right* Overlap is relatively consistent across time points for delta variant.

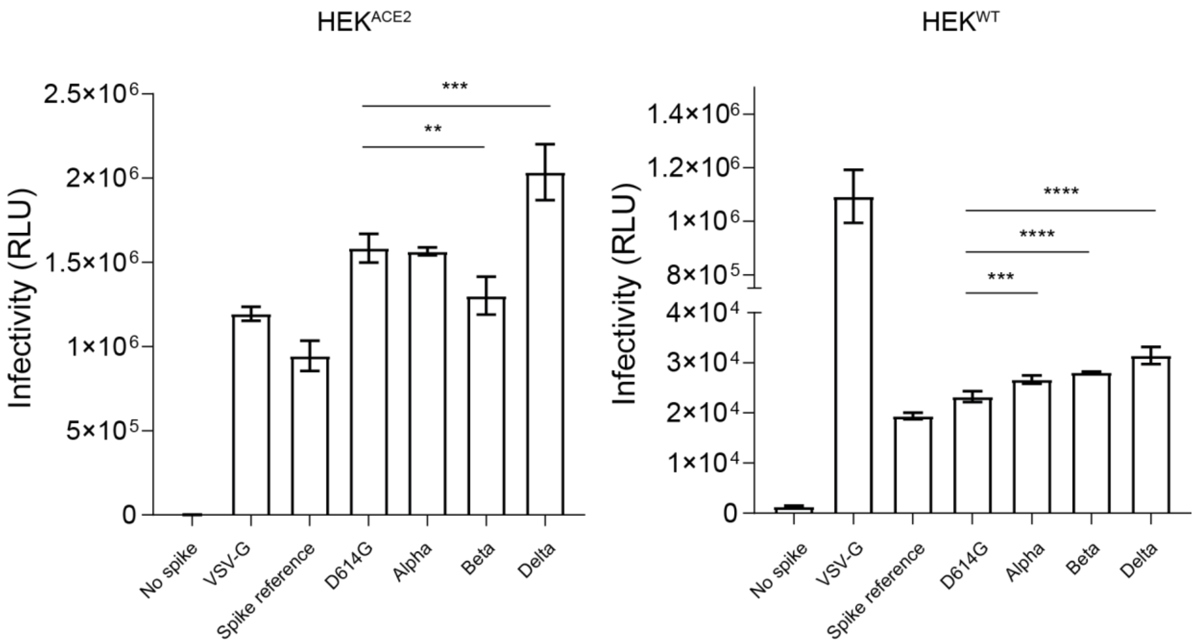

**Supplementary Figure 5:** Infectivity of variant S PVPs. Virus titers (No S, VSV-G, S reference (D614), D614G, Alpha, Beta, and Delta were normalized for RT activity. MOI = 0.2 added to HEK$^{ACE2}$ (*left*) or HEK$^{WT}$ (*right*). Luciferase activity was measured two days post-infection. The experiments was repeated twice with similar results.  ** represents p-value<0.01, *** represents p-value<0.001, and **** represents p-value<0.0001.

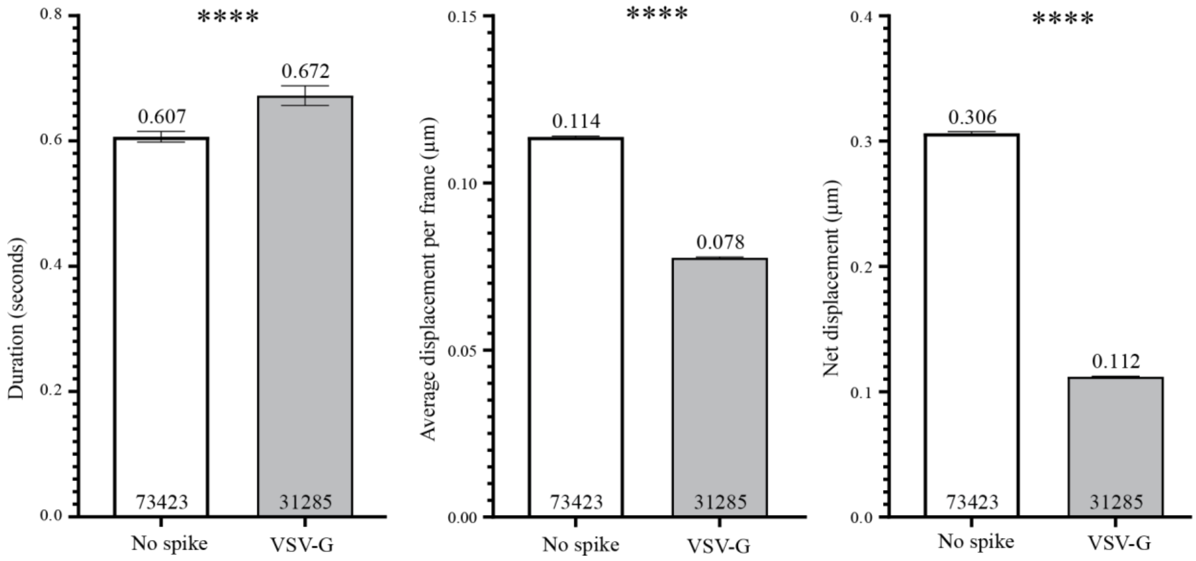


**Supplementary Figure 6:** PVP controls with BEAS-2B cells show duration and displacement trends as expected. VSV-G is able to bind a broad range of cell lines expressing low-density lipoprotein receptor and as such has increased trajectory duration and decreased displacement (both displacement/frame and net displacement) compared to the PVP without a viral glycoprotein.

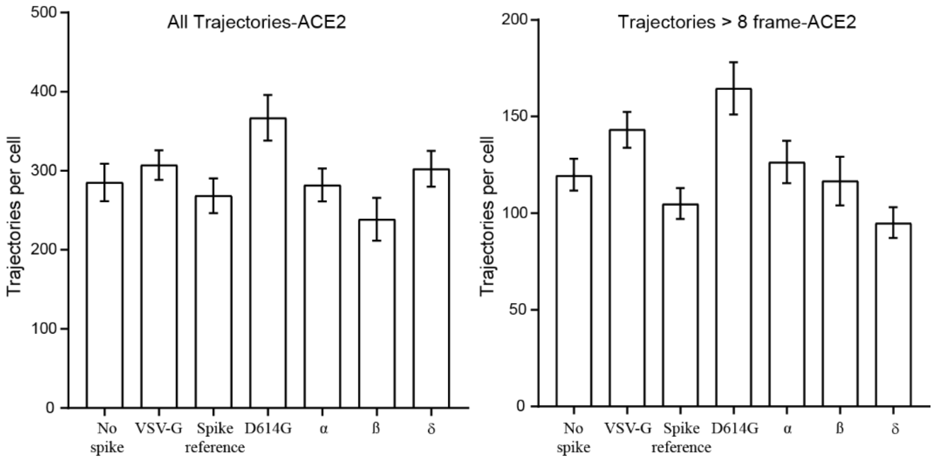


**Supplementary Figure 7:** Trajectories per cell are consistent across S variants. This indicates that the initial diffusion near the cell surface is not dependent on the viral particle glycoprotein, but the glycoprotein does control dynamics of interaction at the cell membrane.

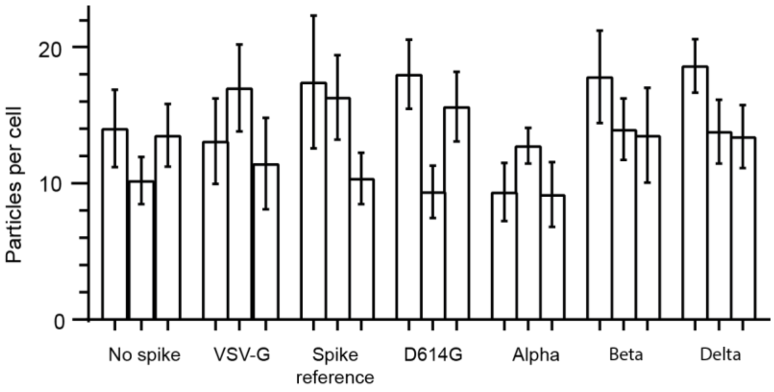


**Supplementary Figure 8:** Particles detected per cell in STORM images is relatively consistent across S variants and time points.

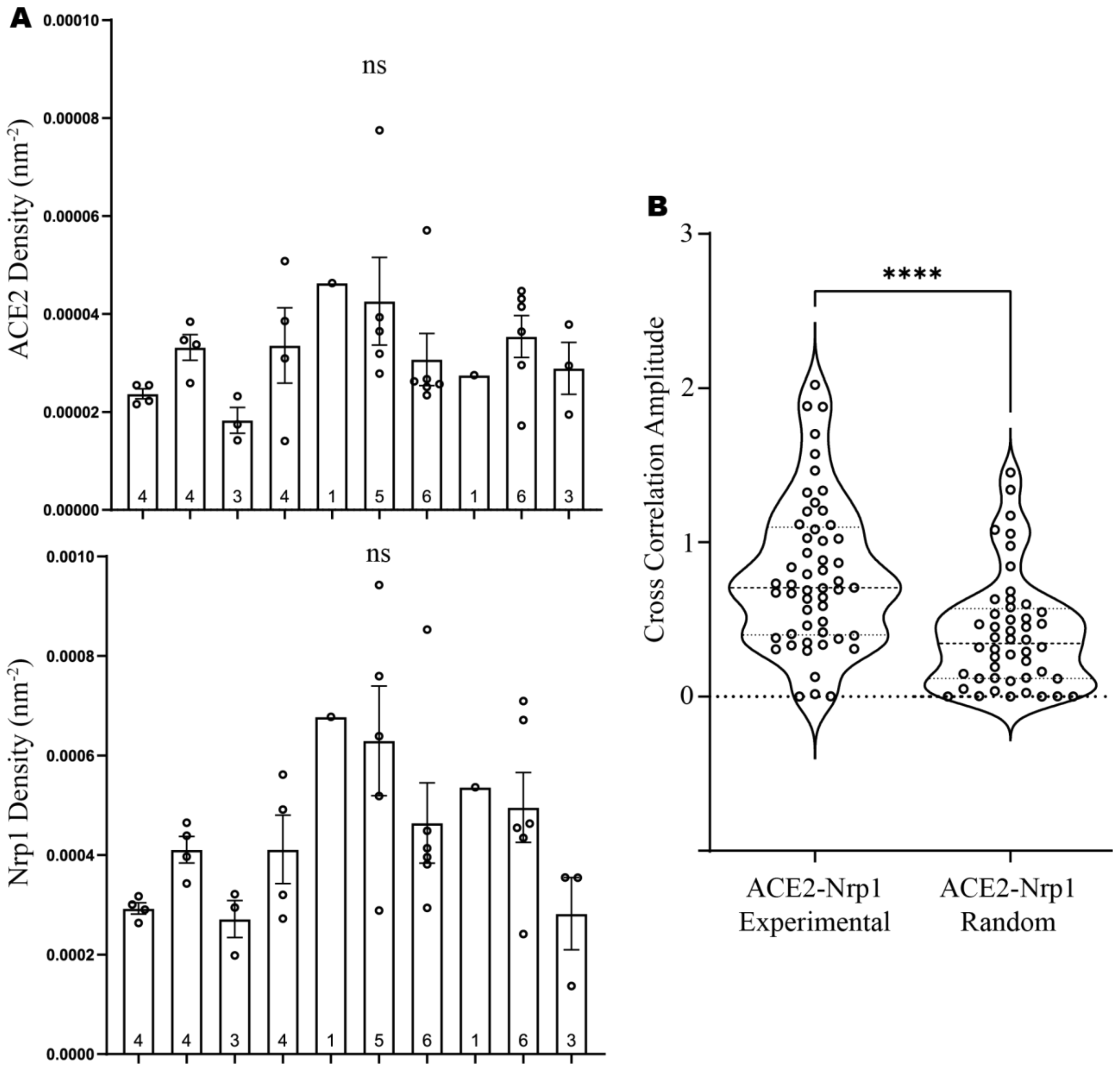


**Supplementary Figure 9: (A)** Receptor density quantified by auto-correlation from a subset of reconstructed cell images. Density of either receptor is not significantly different between cells, with some variability between ROIs of the same cell. **(B)** Receptor colocalization quantified by cross-correlation of experimental and randomized ROIs (n=54). A significant decrease from experimental to randomized ROIs validates that experimental samples have genuine colocalization of the receptors of interest.
